# Introduction to Tobacco Cessation and Motivational Interviewing: Evaluation of a Lecture and Case-Based Learning Activity for Medical Students

**DOI:** 10.7759/cureus.53704

**Published:** 2024-02-06

**Authors:** Daniel J Berger, Sarah Nickolich, Munima Nasir

**Affiliations:** 1 Emergency-Internal Medicine, Virginia Commonwealth University School of Medicine, Richmond, USA; 2 Department of Family and Community Medicine, Penn State College of Medicine, Hershey, USA; 3 Family and Community Medicine, Penn State University College of Medicine, Milton S. Hershey Medical Center, Hershey, USA

**Keywords:** case-based learning, motivational interviewing, nursing, residents, medical students, medical education, smoking cessation, tobacco cessation

## Abstract

Introduction

Despite the fact that tobacco use continues to have significant public health impacts, most healthcare providers are not adequately trained to counsel patients on their tobacco use or to provide cessation resources. Although all healthcare providers have a role in providing tobacco cessation assistance, physicians and advanced practice providers are generally the only practitioners able to furnish tobacco cessation medications and bill insurance for their cessation services. Therefore, ensuring these practitioners are properly trained to offer tobacco cessation to their patients is critical to addressing this public health threat. In line with this goal, this study outlines the curriculum evaluation for an innovative student-facilitated tobacco cessation activity for medical students.

Methods

A lecture and case-based learning activity was created and piloted with a class of first-year medical students. The activity was facilitated by fourth-year medical students. Students took a pre-session survey to establish baseline experience and beliefs and a post-session survey to ascertain their confidence in applying what was covered in the session. Descriptive statistics were utilized to analyze the data.

Results

One hundred and twenty-eight students completed both surveys. Prior to the activity, students reported low levels of confidence in their ability to counsel patients and knowledge of cessation resources. Following the activity, more than 90% reported improvement in their ability to assess a patient’s willingness to quit and counsel those ready to quit. Greater than 80% reported an improvement in their ability to counsel patients not ready to quit and to establish a quit plan. More than 90% of students reported that the session increased their self-efficacy in helping patients quit and that it was worth their time, with 96% committing to increasing their tobacco cessation efforts with their patients.

Discussion

Students valued the training and almost all reported that it increased their ability to help patients quit smoking. The use of student-facilitated case-based learning provided both an opportunity for students to practice cessation techniques and a low-stakes introduction to the OSCE format without the need for extensive faculty resources. Although this session was run with first-year medical students, the curriculum presented can be used for residents, nurses, and other healthcare professionals.

## Introduction

Tobacco use is responsible for a significant health burden, causing approximately 480,000 premature deaths annually in the United States alone [1]. Fortunately, 68.0% of tobacco users report the desire to quit smoking, with 55.4% reporting a quit attempt in the last 12 months [2]. However, among those who attempted to quit smoking, only 31.2% received counseling or tobacco cessation medications, and only 7.4% were successful [2]. Physicians play an important part in encouraging and supporting their patients to quit smoking. However, only 57.2% of tobacco users report being advised by their healthcare provider to quit [2]. Physicians are even less likely to assess patients’ willingness to quit, assist in their quit attempt, or arrange follow-up for their patients’ cessation efforts due to barriers including lack of knowledge, time, and perceived lack of patient motivation [2-4].

Lack of knowledge in how to offer tobacco cessation is not limited solely to practicing physicians. Previous studies have shown that medical students nearing graduation do not feel prepared to help their patients quit smoking [5]. For example, one study, of fourth-year medical students found that although they generally had a good understanding of the epidemiology and risks of tobacco use, only 36% felt adequately or very well prepared to advise their patients to stop smoking and only 15% were aware of cessation programs or services locally available to their patients [5]. Recognizing the need for better tobacco cessation training, the American Association of Medical College’s (AAMC) 2007 Physician Behavior and Practice Patterns Related to Smoking Cessation Summary Report recommended improving the curricula of medical schools and residencies with regard to tobacco control and assisting patients make behavioral changes [3].

Prior to the creation of this curriculum, researchers conducted a needs assessment by surveying students and healthcare providers across our health system regarding their familiarity and comfort with providing various tobacco cessation interventions to patients. Two hundred and eleven pre-clinical and clinic medical students at our institution participated (Survey Data: Berger et al. Healthcare Provider Attitudes Toward and Comfort Providing Smoking Cessation. 2022). Although 83% (n=175) of respondents were very willing to provide non-pharmacologic interventions, including counseling and referrals, only 55% (n=116) were comfortable counseling patients who indicated that they were ready to quit and only 31% (n=65) were comfortable counseling patients not ready to quit. Furthermore, less than a quarter of respondents, including those on clerkships, described themselves as very familiar with tobacco cessation resources such as medications, Quitline, and cessation clinics.

Based on this needs assessment, the authors identified the need for a brief training program that would improve medical student readiness to encourage and support patients in tobacco cessation. As previous studies on the use of simulation-based training for medical students have shown higher rates of participation in similar scenarios during clinical training, we elected to create a paired lecture and case-based learning activity [6].

## Materials and methods

Study design

This study used a pre-/post-single-group study design to evaluate a new one-session training program designed for medical students and other healthcare staff that aimed to increase the quantity and quality of tobacco cessation interventions provided to patients receiving care in inpatient and outpatient settings.

Description of setting and participants

This training was offered as part of the Fundamentals of Patient-Centered Care (FPCC) curriculum, which is the Penn State College of Medicine's longitudinal clinical skills course. The FPCC course revolves around the medical students' “anchor day,” with students assigned to attend the course on either Tuesdays or Wednesdays. All first-year medical students at our institution present on their anchor day when the tobacco cessation FPCC session was offered in December 2022, participated in the activity. The majority of these students had limited clinical experience and had not yet participated in an Observed Structured Clinical Skills Examination (OSCE).

Description of tobacco cessation training curriculum

The one-session training comprised an approximately 45-minute lecture immediately followed by a one-hour case-based learning activity. Due to scheduling limitations, the lectures and cases were conducted virtually using Zoom (Zoom Video Communications, San Jose, USA). The lecture covered the rationale for encouraging patients to quit smoking, an overview of tobacco cessation resources, and an introduction to motivational interviewing techniques (A video slide deck of the lecture slides is found in Video 1 [Appendices]).

The lecture was immediately followed by a one-hour case-based learning activity. To promote a comfortable learning environment, the case-based learning activity was conducted with existing advising groups of 9-10 medical students who were paired with a fourth-year medical student facilitator who acted as both the standardized patient and led the post-case debriefing. Fourth-year medical students were recruited both from our medical school's student-teaching elective, students as educators, and those interested in tobacco cessation.

Students completed three cases. The first case was a patient undergoing an insurance physical who was ready to quit smoking. This case provided an opportunity for students to use their knowledge of cessation resources to establish a quit plan. The second case involved a patient receiving post-hospitalization follow-up for chest pain who was not ready to quit. Students used this case to practice motivational interviewing techniques. The third case was a return visit from the patient in the first case. This patient had recently relapsed from a quit attempt, giving students a chance to practice building self-efficacy in a patient who has relapsed and to develop a new quit plan. Following each case, the facilitator led a debrief, soliciting participation from the entire group. Facilitators were given a rubric to aid them in providing feedback to the learners (A video slide deck of the cases, student resources, and facilitator guides is found in Video 2 [Appendices].)

Cases were presented using a modified OSCE format. Two to three students participated as a team for each case and were permitted to cycle in and out of the primary role. The remaining students observed the encounter and participated in the debrief. Participating students were provided a face sheet for the patient that included the reason for the visit and specific instructions on what they were supposed to accomplish. Unlike a traditional OSCE, they were not required to write a clinical note or present the patient for this activity. Students were provided with a reference sheet of selected lecture slides as well as our institution’s motivational interviewing-inspired tobacco cessation brochure to review prior to the session. Facilitators were provided with the specific learning objectives for each case, along with a detailed packet that included relevant patient medical history and tobacco use behaviors as part of their case. They were also given a grading rubric that provided an overview of the expectations of the student for them to use during the debrief but were not required to complete it.

Study data

To evaluate the training, participating students completed online pre-activity and post-activity surveys using Research Electronic Data Capture (REDCap). The pre-activity survey, completed at the beginning of the training session, was designed to assess baseline student knowledge and behaviors regarding tobacco cessation. The post-activity survey, completed at the end of the session, was designed to assess how the activity impacted students’ perceived ability to help patients quit smoking. “Test” questions were included in each survey to identify respondents who were not closely reading all the questions so that their answers could be reviewed for possible exclusion from the final analyses. The surveys were completed anonymously, but identifying information was collected in a separate form so that students could receive credit for survey completion.

Statistical methods

Statistical analysis was performed using SAS version 9.4 (SAS Institute, Cary, NC, USA) and consisted of descriptive statistics for the two surveys. Students’ responses on the pre- and post-surveys could not be linked, so pre- and post-survey data were analyzed separately. Because students were not required to answer all questions, the denominators for the percentages varied across questions. For the post-activity survey data, chi-square tests were used to compare learner-perceived effectiveness of the case and lecture sessions in meeting the learning objectives. Qualitative feedback about the training session was collected from students via free-response questions on the survey and from the facilitators during a 30-minute post-activity debrief. This project was classified as quality improvement and institutional policy did not require an IRB submission.

## Results

The pre-activity survey was completed by all students (n=135) and the post-activity survey by 94.8% of students (n=128). However, 12.5% of post-activity survey respondents (n=16) were excluded due to incorrect responses on the “test” question and non-response to fill-in questions. Prior to the training session, students reported low levels of comfort in their knowledge of cessation resources and ability to counsel patients on tobacco cessation. Less than half of students (n=61) had encountered patients who used tobacco and only 12% (n=16) reported prior tobacco cessation training. However, the majority believed tobacco cessation is important to patient health (n=127) and that the training would be valuable (n=115). Detailed student pre-session survey responses are shown in Table 1.

**Table 1:** Students’ Pre-Session Survey Responses

Statement	Student Response	n (%)
I feel confident in my ability to counsel patients ready to quit.	Strongly Agree/Agree	36 (26.7)
	Neutral	47 (34.8)
	Strongly Disagree/Disagree	52 (38.5)
I feel confident in my ability to counsel patients not ready to quit.	Strongly Agree/Agree	10 (7.4)
	Neutral	37 (27.4)
	Strongly Disagree/Disagree	88 (65.2)
I have a good understanding of medications and resources to offer patients for tobacco cessation.	Strongly Agree/Agree	13 (9.6)
	Neutral	36 (26.7)
	Strongly Disagree/Disagree	86 (63.7)
I believe tobacco cessation is important for my patients' health.	Strongly Agree/Agree	127 (94.1)
	Neutral	6 (4.4)
	Strongly Disagree/Disagree	2 (1.5)
When I have a patient who uses tobacco, I explicitly advise them to quit.	All/Most of the time	30 (22.2)
	Some of the time	30 (22.2)
	Rarely/Never	17 (12.6)
	I have not encountered a patient who uses tobacco.	58 (43.0)
When I encounter a patient who uses tobacco, I counsel them on reasons and strategies to quit.	All/Most of the time	26 (19.3)
	Some of the time	32 (23.7)
	Rarely/Never	15 (11.9)
	I have not encountered a patient who uses tobacco.	61 (45.2)
Previous training on tobacco cessation or motivational interviewing	Yes	16 (11.9)
	No	119 (88.2)
I believe this training will be valuable.	Strongly agree/agree	115 (85.8)
	Neutral	17 (12.7)
	Strongly disagree/disagree	2 (1.5)

Following the training session, almost all students reported that both the cases and lecture improved their ability to assess, counsel, and assist patients in tobacco cessation. Greater than 90% of students reported that the lecture (n=102) and cases (n=104) were worth their time with 96% (n=108) committing to increasing their tobacco cessation efforts with their patients. Eighty-one percent of students (n=91) who completed the post-session survey reported actively participating in at least one of the three cases. There were no statistically significant differences in responses to the case-based learning outcomes between students who participated in the roleplay versus those who were observers. Detailed post-session survey responses are shown in Table 2.

**Table 2:** Students’ Post-Session Survey Responses

Statement	Student Response	Lecture n (%)	Cases n (%)
Improved my ability to assess willingness to quit	Strongly agree/agree	108 (96.4)	102 (91.1)
	Neutral	3 (2.7)	10 (8.9)
	Strongly disagree/disagree	1 (0.9)	0 (0.0)
Improved my ability to counsel patients ready to quit	Strongly agree/agree	108 (96.4)	108 (96.4)
	Neutral	4 (3.6)	3 (2.7)
	Strongly disagree/disagree	0 (0.0)	1 (0.9)
Improved my ability to counsel patients not ready to quit	Strongly agree/agree	95 (84.8)	92 (82.1)
	Neutral	14 (12.5)	14 (12.5)
	Strongly disagree/disagree	3 (2.7)	6 (5.4)
Improved my ability to establish a quit plan	Strongly agree/agree	98 (87.5)	96 (86.5)
	Neutral	13 (11.6)	12 (10.8)
	Strongly disagree/disagree	1 (0.9)	3 (2.7)
Improved my ability to discuss cessation medications	Strongly agree/agree	84 (75.0)	90 (80.4)
	Neutral	23 (20.5)	15 (13.4)
	Strongly disagree/disagree	5 (4.5)	7 (6.3)
Improved my knowledge of cessation resources	Strongly agree/agree	100 (89.3)	95 (84.8)
	Neutral	12 (10.7)	13 (11.6)
	Strongly disagree/disagree	0 (0.0)	4 (3.6)
Improved my self-efficacy to help patients quit	Strongly agree/agree	104 (92.9)	102 (91.1)
	Neutral	8 (7.1)	9 (8.0)
	Strongly/disagree	0 (0.0)	1 (0.9)
Improved my belief in the importance of tobacco cessation	Strongly agree/agree	93 (83.0)	91 (81.3)
	Neutral	19 (17.0)	20 (17.9)
	Strongly disagree/disagree	0 (0.0)	1 (0.9)
This session was worth my time	Strongly agree/agree	102 (91.1)	104 (92.3)
	Neutral	8 (7.1)	6 (5.4)
	Strongly disagree/disagree	2 (1.8)	2 (1.8)

Student feedback

Reviewing the students’ qualitative feedback, several themes emerged. Students appreciated the framework of the lecture, particularly that it outlined an approach to assess a patient’s willingness to quit and provided strategies based on the patient’s response. Many valued learning about the number of attempts the average patients makes before quitting, the importance of being non-judgmental, and information on cessation medications and resources. Students indicated that they would have liked the lecture to be more interactive and to have included a demonstration of motivational interviewing. They also requested more information on cessation medications.

With regard to the cases, students found the hands-on practice and ability to apply what they learned in the lecture useful, with many commenting on the realism of the cases. They also commented on the value of the differences in the cases, both regarding patient demographics and stage of readiness to quit. Students also noted the quality of the feedback from the fourth-year facilitator and the utility of observing their peers who were participating in the roleplays. Students did suggest using smaller groups in the future so that all students could actively participate in the cases. Some also suggested having a more challenging motivational interviewing case where the patient is more resistant to student attempts.

Facilitator feedback

Feedback from the facilitator debrief indicated that the activity was well received by the students and that the students collectively improved over subsequent cases. One facilitator noted that the training session provided an opportunity for students to practice their communication skills, reporting that students would often make a comment, understand that it did not convey their intended message, and restate it. All reported good participation in the debrief sessions from their group members. Facilitators also noted that the activity provided an orientation to the OSCE format which most students had not yet been exposed to.

## Discussion

Overall, students felt that the pilot curriculum was a valuable use of their time and led to increased self-efficacy regarding their ability to advise and support patients in tobacco cessation. Students indicated a desire for the lecture portion of the training to have more opportunities for interaction, demonstrations of motivational interviewing techniques, and more information on cessation medications. Students appreciated the realism and variety of the cases, although many said that they wished all students could have participated in the roleplay. While the cases were originally intended to be conducted with smaller (four to six student) groups, limited facilitator availability necessitated the pilot using larger student groups. However, the lack of statistically significant differences in feedback from those who actively participated in the roleplays compared to those who were observers indicates that observing the roleplay and then participating in the debriefing provided similar value with regard to learning outcomes.

The use of a fourth-year medical student facilitator enables this training session to be run with less resources than are described in other curricula, as it does not necessitate the use of professional standardized patients or clinician facilitators. Many students identified the value of feedback from the fourth-year facilitator. Previous research on feedback has shown that the credibility of the person providing feedback is an important consideration in how that feedback is received by the recipient, with feedback from those considered to have a low level of knowledge or lacking experience often being disregarded [7]. This pattern was also seen in a previous study conducted at the institution [8]. In that study, students also indicated that an additional advantage of using a fourth-year facilitator was that this created a lower stress environment than would have occurred if their faculty adviser were providing feedback.

The combination of lecture and roleplaying to teach tobacco cessation has been previously documented in the literature, including in a 1994 letter published in the Journal of the American Medical Association [9]. Previous studies have evaluated various lecture, self-directed, and roleplaying curricula with first-year and third-year medical students, reporting that students generally found the various curricula helpful and that participation increased their knowledge and confidence in counseling patients on tobacco cessation [10-13]. Leong et al. reported that students who completed a tobacco cessation curriculum were more likely to offer tobacco cessation to patients in their clerkships and to greater numbers of patients [13].

The students who participated in this curriculum pilot were in their first year of medical school and had little or no patient-care experience. A similar study by Kosowicz et al. reported that students trained in tobacco cessation counseling during their first year of medical school and provided with reinforcement during their third year retained their skills during their fourth year of medical school, suggesting value in training first-year medical students [14]. Utilization of this curriculum for first-year medical students had the additional benefit of orienting students to the OSCE format in a lower stake environment than their first formative OSCE. For example, students were able to practice reviewing a face sheet and initiating a visit with a standardized patient. However, they were also able to rotate with their peer if they were unsure of how to continue in the case and to ask the facilitator questions during the debrief. Thus, this curriculum could be utilized both to orient students for upcoming OSCEs and supplement their clinical experience in motivational interviewing and tobacco cessation.

Similar to this study, students in previous studies have reported increased confidence, knowledge, and use of tobacco cessation counseling skills after training [10-13]. However, not all studies demonstrated proficiency when compared with a professional tobacco cessation counselor [13]. White et al. noted that students could effectively utilize specific questions in the context of motivational interviewing, but they were not always able to effectively engage with the patient’s responses to continue the motivational interviewing process, suggesting that a more limited use of scripted motivational interviewing style questions may be beneficial. Although the data presented is from a pilot of first-year medical students, the brief cessation intervention, resources, and introduction to motivational interviewing covered by the curriculum could easily be adapted for use with medical residents, nurses, or other healthcare professionals, as we have done at our institution.

Limitations

The feedback data presented represents a single class of medical students with limited patient-care experience at a single institution. Although these findings are consistent with those presented in the literature, it is possible that the curriculum may perform differently with other groups of students and in other settings. Existing literature suggests that these students are likely to incorporate these skills with real patients during their clerkships, however, our evaluation was limited to data collected immediately following the session. Further studies are needed to evaluate the effectiveness of training first-year medical students in increasing the frequency and quality of tobacco cessation interventions offered by them to their patients. Additionally, although the student-facilitators were provided a copy of the lecture and facilitator guides for the session, they were not formally trained on tobacco cessation. Therefore, it is possible that student experience differed between facilitator groups. Future studies should consider more rigorous and standardized facilitator training to ensure consistency for learners.

## Conclusions

Tobacco use remains to be a significant public health issue that merits attention from healthcare professionals, necessitating tobacco cessation training for healthcare students. The tobacco cessation lecture and case-based learning activity piloted with first-year medical students were valued by participants, with the vast majority reporting that it increased their ability to help patients quit smoking. The combination of lecture and student-facilitated case-based learning provided an opportunity for students to practice cessation and motivational interviewing without requiring extensive faculty resources while also providing a low-stakes introduction to the OSCE format. Educators should ensure students receive adequate training on tobacco cessation and consider incorporating activities such as the one we present into their curricula.
